# Gastropleural Fistula: A Rare Complication of a Common Procedure

**DOI:** 10.7759/cureus.4136

**Published:** 2019-02-26

**Authors:** Shumaila M Iqbal, Cassandra Zhi, Mawra Masud, Hafiz M Aslam, Madiha A Qadir

**Affiliations:** 1 Internal Medicine, University at Buffalo / Sisters of Charity Hospital, Buffalo, USA; 2 Internal Medicine, Drexel University College of Medicine, Philadelphia, USA; 3 Internal Medicine, Hackensack Meridian School of Medicine at Seton Hall University, Trenton, USA; 4 Internal Medicine, Jinnah Sindh Medical University, Karachi, PAK

**Keywords:** biliopancreatic diversion, gastropleural fistula, hydropneumothorax

## Abstract

Weight loss surgeries are evident to be highly beneficial in patients with morbid obesity (body mass index (BMI) ≥40.0 kg/m^2^) and severe obesity (BMI between 35.0 and 39.9 kg/m^2^ with co-morbidities). While this results in significant mortality benefit, there is always the possible risk of postsurgical complications. Gastrobronchial and gastropleural fistulas are two rare, post-operative pulmonary complications associated with these surgeries. Our patient is a 54-year-old female who underwent a biliopancreatic diversion with a duodenal switch. A few weeks later, she started developing a cough, fever, and shortness of breath. Computed tomography (CT) chest showed the presence of a loculated right sided hydropneumothorax. A gastrointestinal fluoroscopic contrast study performed showed a large fistula originating from the distal end of the stomach and ending towards the right pleural cavity. The fistula was successfully closed with the endoscopic fulguration of fistulous opening with argon beam coagulation and orthoscopic clipping, resulting in complete obliteration of the fistula tract. The right-sided hydropneumothorax was initially treated conservatively with antibiotics and chest tube drains followed by video-assisted thoracoscopic decortication with chest tube placement. Gastropleural fistula formation is rare but is nonetheless a serious postoperative complication of bariatric procedures and mimics pneumonia clinically. It is, therefore, essential to obtain detailed imaging work-up to rule out fistula formation, which, in turn, can be timely treated without causing further devastating results to the patient.

## Introduction

Weight loss surgeries are considered in patients aged 18-60 years, with morbid obesity, body mass index (BMI) ≥40.0 kg/m^2^,or severe obesity, BMI between 35.0 and 39.9 kg/m^2^, with co-morbidities such as diabetes and other metabolic disorders, cardiorespiratory disease, and severe joint disease, in whom weight loss is expected to improve outcomes from those co-morbidities [1]. There are four common types of weight loss surgery practiced in the United States: Roux-en-Y gastric bypass, laparoscopic adjustable gastric banding, sleeve gastrectomy, and duodenal switch with biliopancreatic diversion [2]. The biliopancreatic diversion procedure is one of the most-effective procedures for the treatment of morbid obesity, especially with BMI ≥ 50 kg/m^2^ [3-4]. However, in a study performed by Biertho et al., weight loss surgeries were found to have very efficient results in terms of weight loss and patient satisfaction post-procedure in patients having a BMI <50 kg/m^2 ^[5].

Early surgical complications associated with biliopancreatic diversion with the duodenal switch procedure include infections associated with the incision site, anastomotic leak causing peritonitis, and stricture formation. Nutritional complications are late in onset and include anemia, secondary hyperparathyroidism, and nutritional deficiencies in fat-soluble vitamins [6-7]. Pulmonary complications associated with biliopancreatic diversion are rare; however, if they occur, they tend to be more serious. Early pulmonary complications may include atelectasis and pulmonary embolism similar to the pulmonary complications seen in other surgical procedures. The more specific pulmonary complications associated with bariatric surgeries include gastro-bronchial (GBF) and gastropleural fistulae (GPF) [8]. GPF is a much less frequently reported complication of bariatric surgery as compared to GBF and the reported cases have been seen mainly after sleeve gastrectomy and gastric bypass surgeries [8-11]. Until now, there has been a single case of gastropleural fistulae reported that was found three years after a duodenal switch. It was treated later on by gastrectomy [12]. This present case could add to the limited data on the postoperative pulmonary complications of GPF after a biliopancreatic diversion with a duodenal switch.

## Case presentation

**Figure 1:**
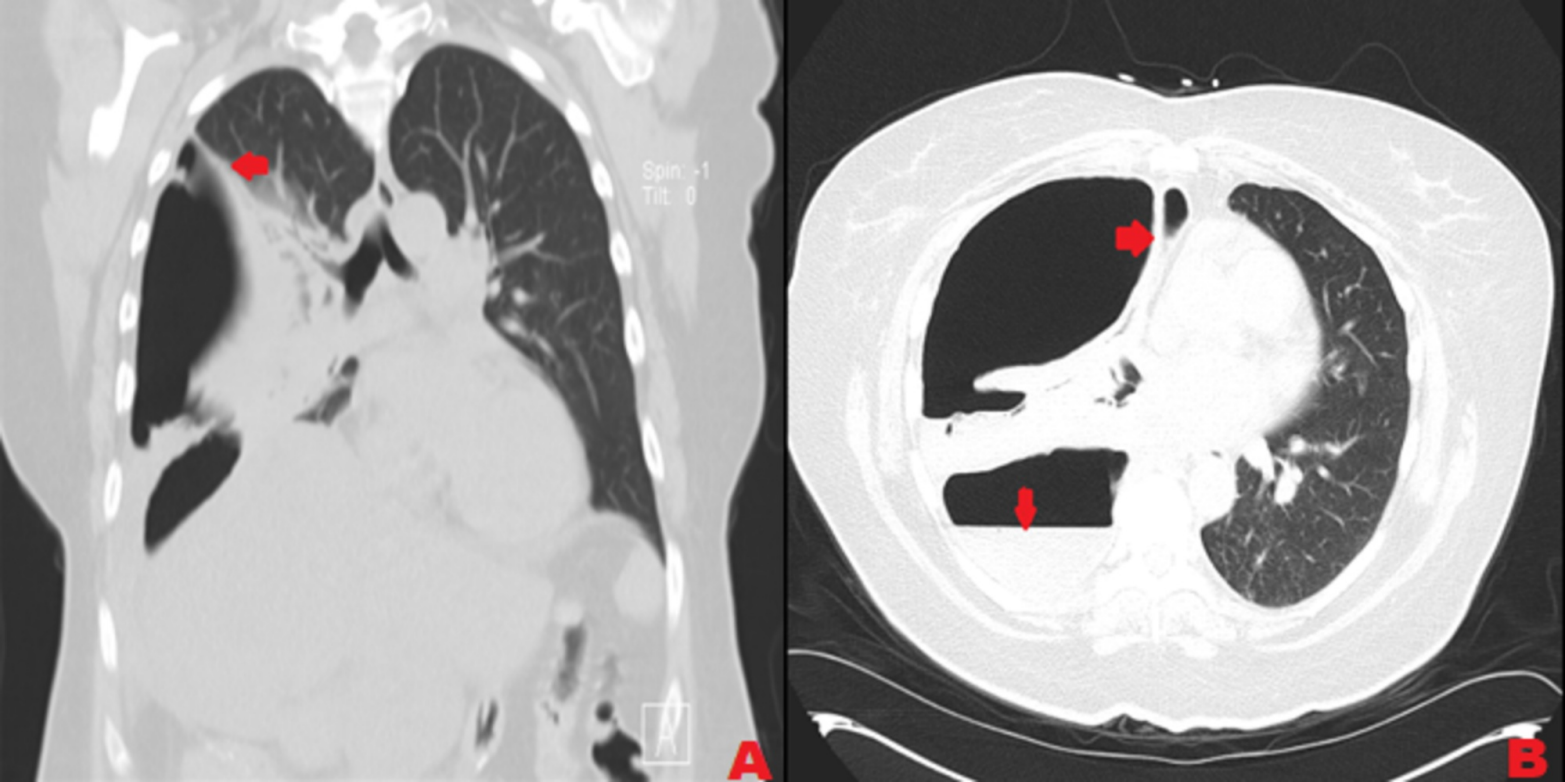
Computed tomography scan of the chest showing right-sided hydropneumothorax with almost complete collapse of the right lung A: Coronal view, B: Axial view

Our patient is a 54-year-old female with a past medical history of hypothyroidism and very severe obesity (BMI 48 kg/m^2^). She underwent laparoscopic gastric sleeve surgery in the year 2012. Results were non-satisfactory in terms of weight loss with a difference of 6 kg/m^2 ^in BMI post-procedure. So after six years, she underwent a laparoscopic biliopancreatic diversion with a duodenal switch. She had an uneventful postoperative recovery period. An upper gastrointestinal (GI) study contrast post-procedure did not reveal any evidence of obstruction or leak. The patient was discharged home two days after the procedure. A few days later, she started experiencing three episodes of nausea with brown-colored vomitus. She was found to be septic, with a heart rate of 110 beats per minute and temperature of 100.2^o^F. Her white blood cells count was 12/mm^3^.The source of infection was presumed to be intraabdominal considering her symptoms. Computed tomography (CT) of the abdomen and pelvis showed mildly dilated proximal small bowel loops. The patient was started on empiric antibiotic therapy with ceftriaxone 1 gm intravenous (IV) daily and metronidazole 500 mg IV every eight hours. Symptoms did not improve, so she was taken back to the operating room for diagnostic laparoscopy. Partial small bowel obstruction was noted along with ischemia of a segment of the ileum that was part of the duodenoileostomy due to mesenteric dissection. She underwent an open revision of the small bowel anastomosis with resection and anastomosis for the obstruction revision of the duodenoileostomy. Her hospital stay post-surgery remained uneventful. Diet was advanced gradually throughout the hospital course and a week later, the patient was discharged home with outpatient follow-up. Three weeks after that procedure, she noticed a productive cough with thick, yellow, foul-smelling phlegm and shortness of breath. She saw her primary care physician. A chest X-ray performed showed a right lung infiltrate with a right-sided pleural effusion. She was started on treatment with augmentin 500 mg/125 mg every eight hours. Her symptoms became worse so she came to the emergency room. Her vitals showed blood pressure 129/79 mmHg, heart rate 86 beats per minute, respiratory rate 20 breaths per minute, and temperature 98.6^o^F. Pulse oxygen saturation was 97% on room air. Mild leukocytosis was evident (white blood cells count 11.4/mm^3^ with no bands or left shift). A chest CT showed loculated, right-sided hydropneumothorax with almost total collapse of the right lung (Figure 1).

**Figure 2:**
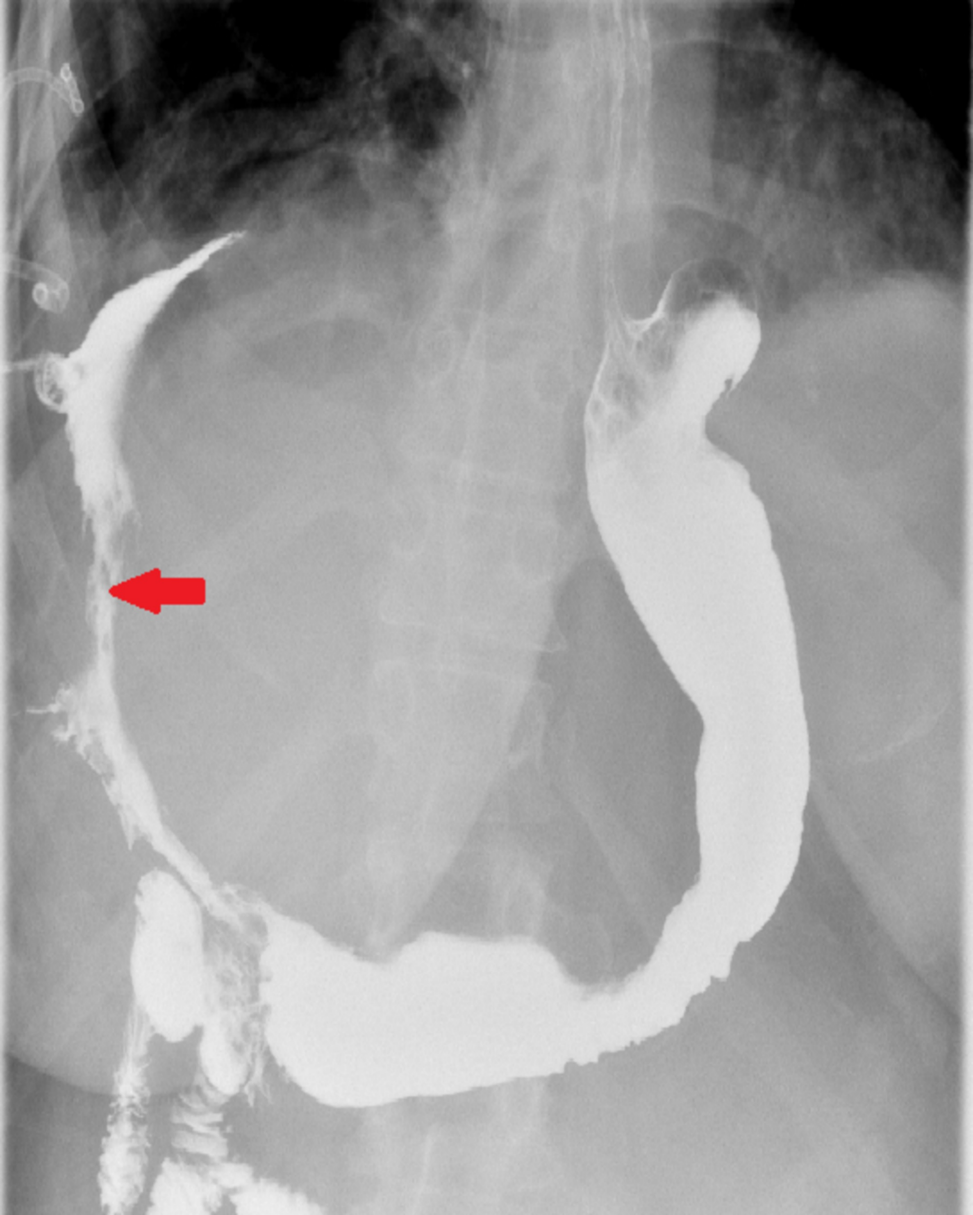
Upper gastrointestinal fluoroscopic contrast study showing fistula formation originating from the distal part of the stomach and terminating in the right pleural cavity

There was a fistulous connection evident, extending from the surgical anastomosis in the stomach/bowel in the right upper quadrant through the right hemidiaphragm to the right hemithorax. These CT scan findings were new as compared to a CT scan obtained for this patient six months prior to the duodenal switch when she presented to the emergency department for non-specific left-sided chest pain. To analyze the anatomy of the fistula further, an upper gastrointestinal fluoroscopic contrast study was performed that showed a large fistula from the distal stomach prior to the duodenal bulb opening to the right pleural cavity (Figure 2).

Consultations from gastroenterology and cardiothoracic surgery teams were obtained. Chest tube drains were placed with the plan of eventually performing a video-assisted thoracoscopic surgical decortication. Post-procedure CT showed patent chest tubes draining the right pleural cavity. The drained fluid was exudative in nature as per Light’s criteria (fluid lactate dehydrogenase > 12,000 u/L and total protein ratio = 0.7) and culture from the right lung empyema grew Escherichia coli, Klebsiella pneumoniae, Pseudomonas aeruginosa, and Candida albicans. An infectious disease consultation was placed at this time. The patient was started on levofloxacin 750 mg IV daily for two weeks as per the sensitivity result obtained for Escherichia coli, Klebsiella pneumoniae, and Pseudomonas aeruginosa. Micafungin 100 mg IV daily was started for the infection with Candida albicans. This was later switched to Diflucan 400 mg IV daily for a total of two weeks. Repeat cultures from the draining fluid were negative toward the end of the second week.

**Figure 3:**
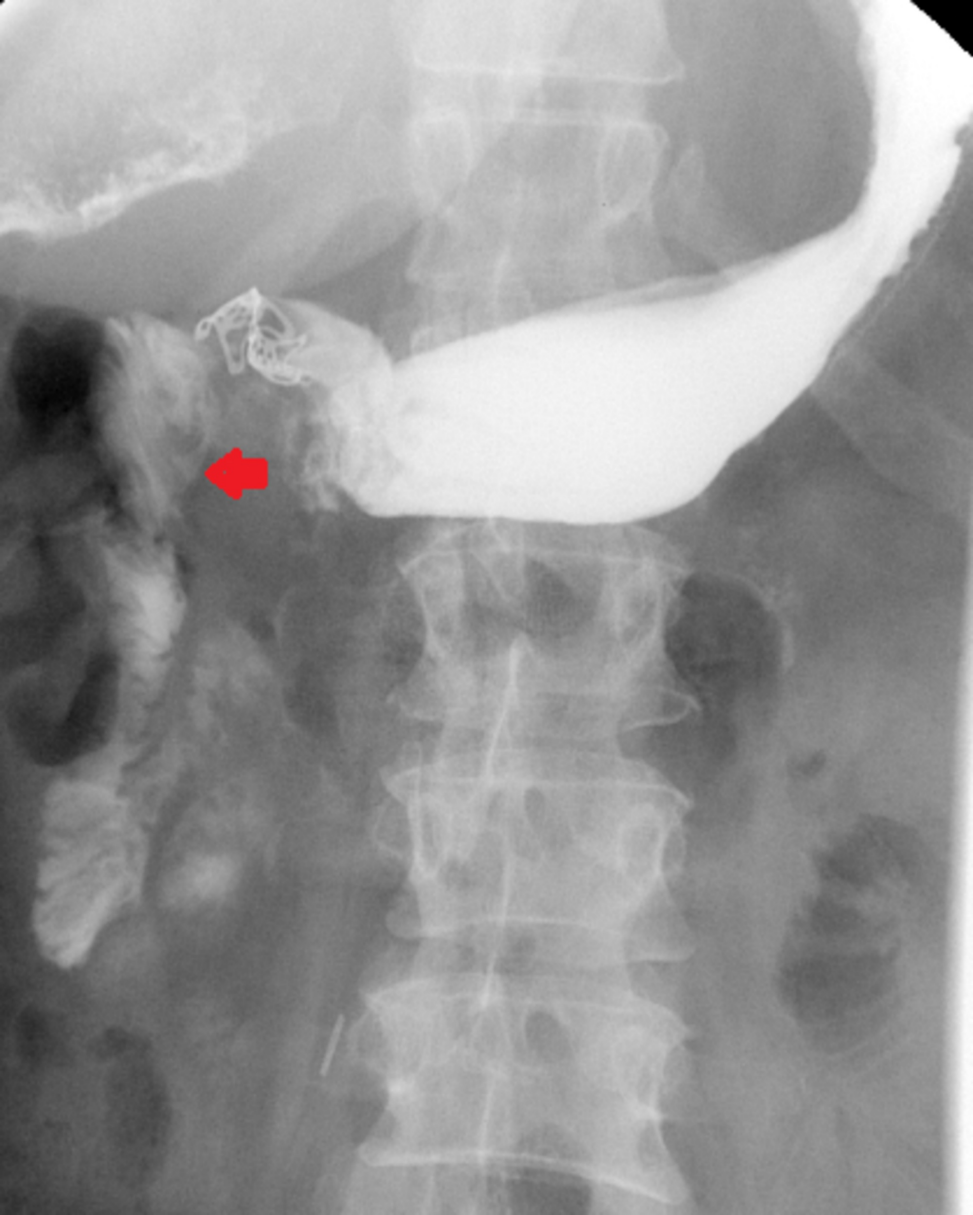
Repeat upper gastrointestinal fluoroscopic contrast study performed post-argon beam coagulation of the fistula showed complete obliteration of the fistula tract

For treatment of the fistula, the patient was transferred to another facility for esophagogastroduodenoscopy (EGD) and possible clipping of the fistula due to the unavailability of that particular service in our hospital. As a result, there was a delay of 16 days from admission to the treatment of the fistula. When the EGD was performed, it showed that there was no anastomotic leak from the previous surgery. No evidence of any stricture was identified at the previous anastomosis. A small fistulous tract was noted in the distal part of the antrum likely secondary to ulcer formation that was noted in very close proximation to the fistula tract. An endoscopic clipping was performed. A post-procedure contrast study performed on the same day revealed complete closure of the fistulous opening. The patient was transferred back to our facility after the procedure. An upper GI contrast study performed two days later showed residual leakage from the distal stomach to the right upper quadrant. A repeat EGD was performed along with fulguration of a fistulous opening with argon beam coagulation and repeat orthoscopic clip application with complete obliteration of the fistula tract. This was confirmed by an upper gastrointestinal contrast study showing no persistent fistulous communication between the post-bulbar duodenum and pleural space (Figure 3).

After confirmation of fistula obliteration, a video-assisted thoracoscopic surgery was performed followed by the washout of the right pleural space with the placement of a right-sided chest tube. The patient was discharged home after the procedure and has been followed by multidisciplinary teams on an outpatient basis.

## Discussion

A gastroparietal fistula is an acquired anatomical communication between the stomach and pleural cavity. They are very uncommon and can occur as a consequence of pulmonary surgeries (resection), intrathoracic gastric perforation in a hiatal hernia, a perforated malignant gastric ulcer at the fundus, extension of a subphrenic abscess with gastric perforation, gastric bypass procedures, or malignancy (Ewing sarcoma) [12-15]. It has also been reported as a complication post-treatment with radiotherapy and chemotherapy [15-16]. They are difficult to manage conservatively and often require gastrectomy and thoracotomy in debilitated patients.

The mechanism for the formation of a gastric fistula after bariatric procedures is not very clear. However, current literature has documented post-operative abscess development at the leak site and subsequent fistula formation [11,17]. In our case, no anastomotic leak was identified and the presence of an ulcer was identified as the cause of fistula formation toward the distal end of the antrum. A GPF can be an early or late complication of bariatric surgery and has been reported to present as late as 13 years post-procedure [8] or as early as three months post-procedure [17]. For our patient, symptoms started appearing four weeks after the duodenal switch procedure. Thus, a rough time frame for the development of a GPF could not be accurately estimated and could happen within a few weeks to a few years after a bariatric procedure.

The clinical presentation for GPF remains insidious and most patients present like our patient, with productive cough, fever, shortness of breath, or chest pain, and radiological studies, including CT scan and X-ray chest, confirming the presence of empyema only [8,17]. Considering the possibility of GPF formation in patients having past bariatric surgery, a contrast oral study should never be missed in order to rule out the possibility of the occurrence. This helps prevent further delay for definitive treatment and the prevention of grave complications.

The prolonged use of antibiotics, total parental nutrition, and chest tube drains can result in the successful management of fistula complications. However, definitive treatment is required either with surgical intervention, such as gastrectomy, thoracotomy, or the endoscopic approach. Endoscopic techniques, such as clipping of the fistula, argon plasma coagulation to induce inflammatory fibrosis with subsequent closure of the tract, with or without the application of Tiessel fibrin glue (Tiessel, Baxter, USA), and the application of a multilayered vicryl mesh can also be used [8,17-18]. In our case presentation, a failed trial of endoscopic clipping resulted in a second EGD with argon plasma coagulation of the fistula tract. This resulted in no further fistula leak on post-EGD fluoroscopic oral contrast study.

## Conclusions

A GPF is a rare but serious complication of almost all forms of bariatric surgeries. This should be considered a possibility in patients presenting with recurrent pneumonia infections or empyema who had bariatric procedures performed in the past, no matter how long ago the initial procedure was performed. Appropriate diagnostic images, such as fluoroscopic oral contrast, should be obtained, as X-ray or CT chest may mask the presence of a fistula. Surgical intervention is usually required. However, the role of endoscopic treatment cannot be minimized. Timely diagnosis and subsequent intervention to close the fistula is key to preventing serious complications.
